# Circulating C19MC MicroRNAs in Preeclampsia, Gestational Hypertension, and Fetal Growth Restriction

**DOI:** 10.1155/2013/186041

**Published:** 2013-11-14

**Authors:** Ilona Hromadnikova, Katerina Kotlabova, Marketa Ondrackova, Andrea Kestlerova, Veronika Novotna, Lucie Hympanova, Jindrich Doucha, Ladislav Krofta

**Affiliations:** ^1^Department of Molecular Biology and Cell Pathology, Third Faculty of Medicine, Charles University, Ruska 87, 100 00 Prague, Czech Republic; ^2^Institute for the Care of the Mother and Child, Third Faculty of Medicine, Charles University, Podolske Nabrezi 157/36, 147 00 Prague, Czech Republic; ^3^Clinic of Obstetrics and Gynecology, Second Faculty of Medicine, Charles University, V Uvalu 84, 150 06 Prague, Czech Republic

## Abstract

The objective of the study was to identify the profile of circulating C19MC microRNAs (miR-516-5p, miR-517∗, miR-518b, miR-520a∗, miR-520h, miR-525, and miR-526a) in patients with established preeclampsia (*n* = 63), fetal growth restriction (*n* = 27), and gestational hypertension (*n* = 23). We examined the correlation between plasmatic concentrations and expression levels of microRNAs and the severity of the disease with respect to clinical signs, requirements for the delivery, and Doppler ultrasound parameters. Using absolute and relative quantification approaches, increased extracellular C19MC microRNA levels (miR-516-5p, *P* = 0.037, *P* = 0.009; miR-517∗, *P* = 0.033, *P* = 0.043; miR-520a∗, *P* = 0.001, *P* = 0.009; miR-525, *P* = 0.026, *P* = 0.01; miR-526a, *P* = 0.03, *P* = 0.035) were detected in patients with preeclampsia. The association analysis pointed to no relationship between C19MC microRNA plasmatic concentrations and expression profile and identified risk factors for a poorer perinatal outcome. However, the dependence between the levels of plasmatic C19MC microRNAs and the pulsatility index in the middle cerebral artery and the values of cerebroplacental ratio was demonstrated. The study brought the interesting finding that the upregulation of miR-516-5p, miR-517∗, miR-520a∗, miR-525, and miR-526a is a characteristic phenomenon of established preeclampsia.

## 1. Introduction

Normal pregnancy is associated with a systemic inflammatory response. Many of the physiologic changes of normal pregnancy are part of an acute-phase reaction, which is generated by an inflammatory response. The placenta is the proximal cause of these problems [1]. Since the placenta is being continuously remodelled during normal placental development, extracellular nucleic acids of both fetal and placental origin, packed into either trophoblast-derived apoptotic bodies or shedding syncytiotrophoblast microparticles, may be detected in maternal circulation during the course of normal gestation [2–6].

Circulating syncytiotrophoblast debris has been hypothesized to contribute to maternal inflammation and some of the features of the maternal syndrome [7]. Signs of maternal inflammation which appear to be present in normal pregnancies at term are exaggerated in preeclampsia (PE) and fetal growth restriction (FGR) and can account for their clinical features [1, 8].

Preeclampsia and fetal growth restriction (FGR) are major complications affecting about 2–10% of pregnancies responsible for maternal and perinatal morbidity and mortality [9, 10]. Preeclampsia usually develops after 20 weeks of gestation and is characterized by chronic or gestational hypertension combined with proteinuria, which results from defective placentation eliciting inadequate uteroplacental blood perfusion and ischemia [8, 11, 12]. The causes of preeclampsia and FGR remain unknown. Trophoblastic debris and the microparticles shed during normal pregnancy are proinflammatory and this process is amplified in preeclampsia [13]. A hypoxic environment induces excessive trophoblast cell death and increased shedding of placenta debris into the maternal circulation; as a result, placental insufficiency related pregnancy complications are associated with abnormal levels of extracellular fetal DNA and mRNA transcripts [5, 14].

Recent evidence suggests that preeclampsia can be further subdivided into early PE (before 34 weeks of gestation), intermediate PE (between 34 and 37 weeks of gestation), and late PE (after 37 weeks of gestation) [15, 16]. The concept of early and late PE is modern, and it is widely accepted that these two entities have different etiologies and should be regarded as different forms of the disease, where early onsets of PE and IUGR are considered to be placenta-mediated diseases [17–19].

There has been a trend over the last 10 years to develop noninvasive methods utilizing quantification of cell-free nucleic acids inclusive of microRNAs in maternal circulation [6, 20–39]. The diagnostic potential of particular molecular biomarkers and their implementation in the current predictive and diagnostic algorithms for pregnancy related complications is subject of interest [6].

MicroRNAs belong to a family of small noncoding RNAs that regulate gene expression at the posttranscriptional level by degrading or blocking translation of messenger RNA (mRNA) targets [40, 41]. 

Recent studies have shown that preeclampsia is associated with alterations in extracellular microRNA expression. Using real-time PCR analysis, Gunel et al. [42] demonstrated the upregulation of miR-210 and downregulation of miR-152 in patients with preeclampsia. The application of next generation sequencing technology revealed a broader profile of dysregulated circulating microRNAs in preeclampsia. Compared to controls, 15 microRNAs were found to be upregulated (miR-521, miR-520h, miR-517c, miR-519d, miR-520g, miR-517b, miR-542-3p, miR-136, let-7f-1*, miR-518e, let-7a*, miR-125b, miR-125 a-5p, miR-519a, and miR-29a) and 7 microRNAs were found to be downregulated (let-7f, miR-223, miR-1260, let-7d, miR-320c, miR-185, and miR-1272) in four examined preeclamptic serum samples [43].

Later, using microarray analysis Wu et al. [44] reported the upregulation of 13 microRNAs (miR-574-5p, miR-26a, miR-151-3p, miR-130a, miR-181a, miR-130b, miR-30d, miR-145, miR-103, miR-425, miR-221, miR-342-3p, and miR-24) and down-regulation of 2 microRNAs (miR-144, miR-16) in patients with severe preeclampsia. Seven of these 13 microRNAs (miR-574-5p, miR-26a, miR-130b, miR-181a, miR-342-3p, miR-103, and miR-24) were validated by real-time PCR analysis to be elevated in plasma from severe preeclamptic pregnancies.

In a small-scale analysis, Mouillet et al. [45] did not observe any differentiation between pregnancies with normal and fetal growth restricted fetuses when compared circulating microRNA expression levels (miR-27a, miR-30d, miR-141, miR-200c, miR-205, miR-424, miR-451, miR-491, miR-517a, miR-518b, miR-518e, and miR-524).

However, most of investigators focused on the study of those microRNAs, whose genes are located outside chromosome 19 miRNA clusters (C19MC and miR-371-3 cluster) or the chromosome 14 miRNA cluster (C14MC) that encode pregnancy-associated microRNAs [46–50].

We have previously identified C19MC microRNAs (miR-516-5p, miR-517*, miR-518b, miR-520a*, miR-520h, miR-525, and miR-526a) present in maternal plasma differentiating between normal pregnancies and nonpregnant individuals [51]. We selected from the chromosome 19 microRNA cluster, which involves 46 microRNA genes altogether, [48–50, 52] preferentially those microRNAs that were previously demonstrated to be exclusively expressed in placental tissues (miR-520a*, miR-516-5p, miR-517*, miR-518b, miR-519a, miR-524-5p, miR-525, miR-526a, miR-526b, and miR-520h) and those microRNAs that were reported to be highly expressed in placental tissues (miR-512-5p, miR-515-5p, miR-518f*, miR-519d, and miR-519e*) [51, 53, 54].

Later, we demonstrated significant increases in extracellular C19MC microRNAs levels (miR-516-5p, miR-517*, miR-518b, miR-520a*, miR-520h, miR-525, and miR-526a) over time in normally progressing pregnancies [51, 54].

The results of our pilot study indicated no differentiation between normal and complicated pregnancies, but could not come to definitive conclusions due to the low number of studied subjects involved [51, 54]. The current study is a followup of our previous studies [51, 54] and describes comprehensively for the first time the expression profile of circulating C19MC microRNAs (miR-516-5p, miR-517*, miR-518b, miR-520a*, miR-520h, miR-525, and miR-526a) in the entirely new sample set of patients with clinically established preeclampsia and/or fetal growth restriction. To our knowledge, no study describing the profile of circulating C19MC microRNAs in gestational hypertension has been carried out.

## 2. Materials and Methods

### 2.1. Patients

The studied cohort consisted of Caucasian women involving 63 preeclampsia (PE) w or w/o fetal growth restriction (FGR), 27 FGR, 23 gestational hypertension (GH), and 55 controls. Twenty-four women had signs of mild preeclampsia, 39 women were diagnosed with severe preeclampsia, 24 preeclamptic patients required the delivery before 34 weeks of gestation and 39 patients delivered after 34 weeks of gestation. In 18 cases, preeclampsia superposed on previous hypertension; otherwise, it occurred in normotensive patients (45 cases). Eight growth-retarded foetuses were delivered before 34 weeks of gestation and 19 those after 34 weeks of gestation. Oligohydramnios or anhydramnios were present in 7 growth restricted foetuses.

Doppler studies showed an abnormal pulsatility index (PI) in the umbilical artery (14 preeclampsia ± FGR and 14 FGR) and/or in the middle cerebral artery (10 preeclampsia ± FGR and 11 FGR). Cerebroplacental ratio (CPR), expressed as a ratio between umbilical artery and middle cerebral artery pulsatility index, was below the fifth percentile in 21 cases (9 preeclampsia ± FGR and 12 FGR). Absent or reversed enddiastolic velocity waveforms in the umbilical artery occurred in 8 cases (2 preeclampsia + FGR and 6 FGR).

Normal pregnancies were defined as those without complications who delivered full term, singleton, healthy infants weighting >2500 g after 37 completed weeks of gestation. Preeclampsia was defined as blood pressure >140/90 mmHg in two determinations 4 hours apart that was associated with proteinuria >300 mg/24 h after 20 weeks of gestation. Severe preeclampsia was diagnosed by the presence of one or more of the findings according to the guidelines of ACOG Committee [11].

Fetal growth restriction was diagnosed when the estimated fetal weight (EFW), calculated using the Hadlock formula (Astraia Software GmbH), was below the tenth percentile for the evaluated gestational age.

Gestational hypertension was defined as high blood pressure that developed after the twentieth week of pregnancy.

All patients provided written informed consent. The study was approved by the Ethics Committee of the Third Faculty of Medicine, Charles University in Prague. The samples for the study were chosen on the basis of equal times in storage and gestation age. Gestational age was assessed using ultrasonography.

### 2.2. Processing of Samples

Nine millilitres of peripheral blood were collected into EDTA tubes and centrifuged twice at 1200 g for 10 min at room temperature. Plasma samples were stored at −80°C until subsequent processing. 

Total RNA was extracted from 1 mL of plasma and 25 mg of normal placental tissue preserved in RNAlater (Ambion, Austin, USA) followed by an enrichment procedure for small RNAs using a mirVana microRNA Isolation kit (Ambion, Austin, USA). Trizol LS reagent was used in plasma samples for total RNA extraction from biological fluids (Invitrogen, Carlsbad, USA) and preceded the small RNAs enrichment procedure. To minimize DNA contamination, we treated the eluted RNA with 5 *µ*L of DNase I (Fermentas International, ON, Canada) for 30 min at 37°C.

### 2.3. Reverse Transcriptase Reaction

Each microRNA was reverse-transcribed into complementary DNA using TaqMan MicroRNA Assay, containing microRNA-specific stem-loop RT primers (Table 1), and TaqMan MicroRNA Reverse Transcription Kit (Applied Biosystems, Branchburg, USA) in a total reaction volume of 50 *µ*L on a 7500 real-time PCR system (Applied Biosystems, Branchburg, USA) with the following thermal cycling parameters: 30 minutes at 16°C, 30 minutes at 42°C, 5 minutes at 85°C, and then held at 4°C.

### 2.4. Quantification of MicroRNAs

15 *µ*L of cDNA corresponding to each microRNA was mixed with components of TaqMan MicroRNA Assay and the ingredients of the TaqMan Universal PCR Master Mix (Applied Biosystems, Branchburg, USA) in a total reaction volume of 35 *µ*L. TaqMan PCR conditions were set as described in the TaqMan guidelines. The analysis was performed using a 7500 real-time PCR system. All PCRs were performed in duplicates. A sample was considered positive if the amplification signal occurred before the 40th threshold cycle. Concentrations of individual microRNAs were expressed as pg of total RNA enriched for small RNAs per millilitre of plasma. 

The expression of particular microRNA in maternal plasma was determined using the comparative Ct method [55] relative to the expression of the same microRNA in the reference sample, randomly selected placenta derived from gestation with normal course.

RNA fraction highly enriched for small RNA isolated from the fetal part of the placenta (the part of the placenta derived from the chorionic sac that encloses the embryo, consisting of the chorionic plate and villi) was used to build-up the standard curves and as a reference sample for relative quantification throughout the study.

Synthetic C. elegans microRNA (cel-miR-39, Qiagen, Hilden, Germany) was used as an internal control for variations during the preparation of RNA, cDNA synthesis, and real-time PCR. Due to a lack of generally accepted standards, all experimental real-time qRT-PCR data were normalized to cel-miR-39, as it shows no sequence homology to any human microRNA. 1 *µ*l of 0.1 nM cel-miR-39 was spiked in after incubation with Trizol LS reagent to the human plasma samples.

### 2.5. Statistical Analysis

MicroRNA levels were compared between groups by nonparametric tests (the Mann-Whitney *U* test for the comparison between two groups and the Kruskal-Wallis test for the comparison between three or more groups) using Statistica software (StatSoft Inc., USA). Correlation between variables including absolute and/or relative microRNA quantification and Doppler ultrasonography parameters (the pulsatility index in the umbilical artery, the pulsatility index in the middle cerebral artery, and the cerebroplacental ratio) was calculated using the Spearman's rank correlation coefficient (*ρ*). If it varies from −0.5 to 0, there is a weak negative correlation. The significance level was established at a *P* value of *P* < 0.05.

## 3. Results

### 3.1. Circulating C19MC MicroRNAs Differentiate between Complicated and Normal Pregnancies

Overall, increased plasmatic levels of miR-516-5p (*P* = 0.008), miR-517* (*P* = 0.003), miR-520a*. (*P* < 0.001), miR-525 (*P* = 0.003), and miR-526a (*P* = 0.004) were observed in women with pregnancy-related complications (gestational hypertension, preeclampsia and fetal growth restriction) compared to normal pregnancies. 

Similarly, the difference in gene expression of circulating microRNAs between pregnancy-related complications and the control cohort (normal pregnancies) achieves statistical significance for miR-516-5p (*P* < 0.001), miR-517* (*P* = 0.005), miR-520a* (*P* = 0.001), miR-525 (*P* = 0.001), and miR-526a (*P* = 0.004).

### 3.2. Upregulation of Circulating C19MC MicroRNAs in Pregnancies with Established Preeclampsia

Consecutive detailed group analysis confirmed a difference in the levels of extracellular microRNAs in 5/5 C19MC microRNAs (miR-516-5p, *P* = 0.037; miR-517*, *P* = 0.015; miR-520a*, *P* = 0.003; miR-525, *P* = 0.026; and miR-526a, *P* = 0.032).

**Figure 1:**
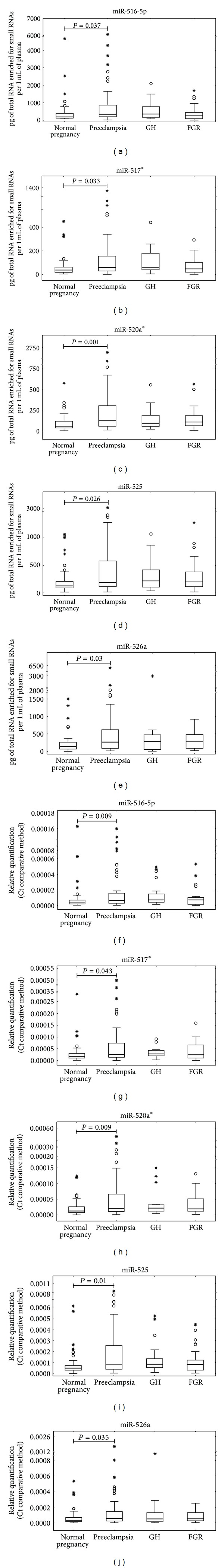
Upregulation of circulating C19MC microRNAs in pregnancies with preeclampsia. Absolute ((a), (b), (c), (d), and (e)) and relative ((f), (g), (h), (i), and (j)) quantification data were expressed as box plots of individual microRNAs in cohorts of normal and complicated pregnancies using Statistica software. The upper and lower limits of the boxes represent the 75th and 25th percentiles, respectively. The upper and lower whiskers represent the maximum and minimum values that are no more than 1.5 times the span of the interquartile range (range of the values between the 25th and the 75th percentiles). The median is indicated by the line in each box. Outliers are indicated by circles and extremes by asterisks.

While plasmatic levels of microRNAs between the control cohort and the cohorts of patients with FGR and GH did not differ, increased levels were detected in the group of patients with established preeclampsia (miR-516-5p, *P* = 0.037; miR-517*, *P* = 0.033; miR-520a*, *P* = 0.001; miR-525, *P* = 0.026; and miR-526a, *P* = 0.030) (Figures 1(a)–1(e)).

Parallel, significant difference in microRNA gene expression was found between groups of preeclampsia, gestational hypertension, fetal growth restriction, and controls (miR-516-5p, *P* = 0.005; miR-517*, *P* = 0.028; miR-520a*, *P* = 0.011; miR-525, *P* = 0.01; miR-526a, *P* = 0.034). Again, while the expression of microRNAs between the control cohort, gestational hypertension, and fetal growth restriction did not differ, the highest expression was detected in the group of patients with preeclampsia (miR-516-5p, *P* = 0.009; miR-517*, *P* = 0.043; miR-520a*, *P* = 0.009; miR-525, *P* = 0.01; miR-526a, *P* = 0.035) (Figures 1(f)–1(j)).

### 3.3. The Association Study of Circulating C19MC MicroRNAs and the Severity of the Disease with respect to Clinical Signs and Requirements for the Delivery

Plasmatic concentrations and/or expression profile of C19MC microRNAs were analysed in relation to the severity of the disease with respect to the degree of clinical signs (mild and severe preeclampsia) and requirements for the delivery (before and after 34 weeks of gestation). No effect of the severity of the disease either on plasmatic C19MC microRNA concentrations (miR-516-5p, *P* = 0.396; miR-517*, *P* = 0.226; miR-520a*, *P* = 0.08; miR-525, *P* = 0.237; and miR-526a, *P* = 0.201) or C19MC microRNA expression levels (miR-516-5p, *P* = 0.476; miR-517*, *P* = 0.58; miR-520a*, *P* = 0.239; miR-525, *P* = 0.397; miR-526a, *P* = 0.646) was observed.

Further, the association between C19MC microRNA plasmatic levels and/or gene expression and the occurrence of previous hypertension in the cohort of patients with preeclampsia was determined. No difference between the group of preeclampsia superposed on chronic hypertension and/or gestational hypertension and the group of patients with unexpected onset of preeclampsia was revealed (absolute quantification: miR-516-5p, *P* = 0.885; miR-517*, *P* = 0.538; miR-520a*, *P* = 0.342; miR-525, *P* = 0.909; miR-526a, *P* = 0.273; relative quantification: miR-516-5p, *P* = 0.721; miR-517*, *P* = 0.621; miR-520a*, *P* = 0.885; miR-525, *P* = 0.568; miR-526a, *P* = 0.201).

### 3.4. The Association Study of Circulating C19MC Micrornas and the Severity of the Disease with respect to Doppler Ultrasonography Monitoring

The association between the plasmatic concentration and gene expression levels of C19MC microRNAs and Doppler ultrasonography parameters (the pulsatility index in the umbilical artery the pulsatility index in the middle cerebral artery, and the cerebroplacental ratio) was studied in the cohort of pregnancies complicated with preeclampsia and/or fetal growth restriction.

No difference within the group of complicated pregnancies with normal and abnormal values of flow rate in the umbilical artery was found out with the exception of miR-526a, which was upregulated in the group of patients with abnormal blood flow velocity waveforms (absolute quantification: *P* = 0.038; relative quantification: *P* = 0.05).

Further, the statistical analysis showed no effect of the pulsatility index in the middle cerebral artery and the cerebroplacental ratio on the plasmatic concentrations (A. cerebri media: *P* = 0.479, *P* = 0.826, *P* = 0.528, *P* = 0.625, *P* = 0.154; CPR: *P* = 0.426, *P* = 0.479, *P* = 0.443, *P* = 0.867, and *P* = 0.181) and expression levels (A. cerebri media: *P* = 0.826, *P* = 0.931, *P* = 0.427, *P* = 0.639, and *P* = 0.297; CPR: *P* = 0.517, *P* = 0.288, *P* = 0.198, *P* = 0.984, and *P* = 0.195) of all microRNAs (miR-516-5p, miR-517*, miR-520a*, miR-525, and miR-526a) that were identified to be upregulated in plasma samples derived from preeclampsia with or without fetal growth restriction.

**Figure 2:**
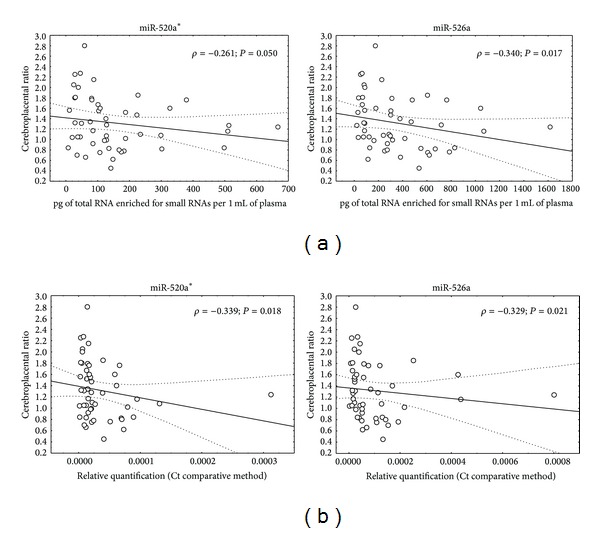
The association between the plasmatic concentration (a) and expression levels (b) of C19MC microRNAs and Doppler ultrasonography parameters. *ρ*: Spearman's correlation coefficient; *P*: level of significance.

The correlation between variables including absolute and/or relative quantification of particular microRNA in maternal plasma and the values of flow rate in the umbilical artery and the fetal blood vessel was calculated using Spearman's rank correlation coefficient. The pulsatility index in the umbilical artery did not show any correlation with microRNA plasmatic concentrations and/or microRNA gene expression. However, a weak negative correlation between the pulsatility index in the middle cerebral artery and microRNA plasmatic concentrations (miR-516-5p :  *ρ* = −0.393, *P* = 0.005; miR-517*: *ρ* = −0.328, *P* = 0.020; miR-520a*: *ρ* = −0.314, *P* = 0.026; miR-525: *ρ* = −0.358, *P* = 0.011; miR-526a: *ρ* = −0.304, *P* = 0.031) or microRNA gene expression (miR-516-5p: *ρ* = −0.307, *P* = 0.030; miR-517*: *ρ* = −0.288, *P* = 0.041; miR-520a*: *ρ* = −0.339, *P* = 0.017; miR-525: *ρ* = −0.357, *P* = 0.012; miR-526a: *ρ* = −0.286, *P* = 0.043) was observed. Furthermore, a weak negative correlation between cerebroplacental ratio and microRNA plasmatic concentrations (miR-520a*: *ρ* = −0.261, *P* = 0.050; miR-526a: *ρ* = −0.340, *P* = 0.017; Figure 2(a)) or microRNA gene expression (miR-520a*: *ρ* = −0.339, *P* = 0.018; miR-526a: *ρ* = −0.329, *P* = 0.021; Figure 2(b)) was found (Table 1).

### 3.5. Function and Functional Relationship Analysis of Target Genes of Differentially Expressed Extracellular C19MC MicroRNAs in Preeclampsia

The function and functional relationship analysis of predicted targets of the five elevated extracellular C19MC microRNAs in patients with established preeclampsia indicated that a large group of genes was connected to the regulation of the immune system and inflammatory response (Table 2). The data were collected from miRDB database (http://mirdb.org/miRDB/). All the targets were predicted by a bioinformatics tool MirTarget2, which was developed by analyzing thousands of genes impacted by miRNAs with an SVM learning machine.

## 4. Discussion

The results of our previous pilot study strongly supported the need for a more detailed exploration of extracellular microRNAs in maternal circulation with the view toward their recognition as potential biomarkers for placental insufficiency related complications [51, 54].

Initially, some extracellular placental specific microRNAs (miR-516-5p, miR-520*, miR-518b, and miR-526a) trended just to a higher level in the small cohort of patients with placental insufficiency related complications (16 preeclampsia, 5 preeclampsia with IUGR and 11 IUGR), however, did not reach statistical significance when compared to gestational-age-matched controls. Although we have previously demonstrated that normal pregnancies and the manifestation of placental-insufficiency-related complications did not influence the levels of miR-16 and let-7d in maternal plasma [51, 54], it is now no doubt that ubiquitously expressed miR-16 and let-7d should not be further used to normalize expression profiles of various extracellular microRNAs as was done before in our pilot study. Our latest research revealed that the expression levels of miR-16 were significantly decreased in placental tissues derived from patients with preeclampsia (data submitted for publication). Similarly, Maccani et al. [56] also reported that reduced expression of miR-16 in placental tissue may be relevant to the low birth weight in term infants born small for gestational age. In contrast, miR-16 was previously observed to be overexpressed in placental tissues affected with severe preeclampsia, respectively [38]. The latest study of Wu et al. [44] brought the evidence of circulating mir-16 down-regulation in patients with severe preeclampsia. Furthermore, Yang et al. [43] showed decreased expression of circulating let-7d in preeclamptic patients applying more sophisticated approach such as next generation sequencing technology.

For that reason, alternative endogenous control candidates to normalize extracellular microRNA gene expression data should be used.

In the current study, the cohort of pregnancy related complications was expanded to achieve adequate power of the study. This time, the normalization of circulating microRNA expression was done against synthetic C. elegans microRNA (cel-miR-39). Using both absolute and relative quantification approaches, the ability of extracellular C19MC microRNAs (miR-516-5p, miR-517*, miR-520a*, miR-525, and miR-526a) to differentiate between normal and complicated pregnancies during the onset of preeclampsia w or w/o fetal growth restriction was confirmed.

Unfortunately, limited data comparing extracellular C19MC microRNA levels between the groups of normal and complicated pregnancies are available. Our data are inconsistent with Yang et al. [43], who observed the upregulation of extracellular miR-520h in four patients with preeclampsia.

Our findings may be supported by Mouillet et al. [45], who have recently also observed no significant difference in relative placental specific microRNA levels (miR-518b) in plasma samples from those with normally progressing and fetal growth restriction pregnancies.

On the basis of the results of our study, we further studied the association between circulating C19MC microRNAs and the severity of the disease with respect to the degree of clinical signs, requirements for the delivery (before and after 34 weeks of gestation), and Doppler ultrasound examination.

The association analysis pointed to no relationship between C19MC microRNA plasmatic concentrations and/or gene expression and identified risk factors for a poorer perinatal outcome. There was no difference in microRNA plasmatic levels and/or gene expression between pregnancies with mild and severe preeclampsia, pregnancy-related complication with the need for the delivery before 34 weeks of gestation, and those who delivered after this critical period and pregnancies with abnormal and normal blood flow velocity waveforms. Nevertheless, the levels of miR-526a were significantly increased in the group of patients with abnormal values of flow rate in the umbilical artery.

On the other hand, the dependence between the levels of plasmatic C19MC microRNAs and the pulsatility index in the middle cerebral artery and the values of cerebroplacental ratio was demonstrated. The relation between the increased levels of plasmatic C19MC microRNAs (miR-516-5p, miR-517*, miR-520a*, miR-525, and miR-526a) and decreased values of flow rate in the middle cerebral artery reached a statistical significance in complicated pregnancies. 

Similarly, the relationship between increased levels of plasmatic C19MC microRNAs (miR-520a* and miR-526a) and decreased values of cerebroplacental ratio was revealed.

In conclusion, microRNAs play a fundamental role in a variety of physiological and pathological processes involving pregnancy-related complications. Current study demonstrated for the first time that circulating C19MC microRNAs might play a role in the pathogenesis of preeclampsia, but not in the pathogenesis of gestational hypertension and fetal growth restriction. The study brought interesting finding that the upregulation of circulating C19MC microRNAs (miR-516-5p, miR-517*, miR-520a*, miR-525, and miR-526a) is a characteristic phenomenon of established preeclampsia.

## 5. Conclusion

The study brought the interesting finding that the upregulation of circulating C19MC microRNAs (miR-516-5p, miR-517*, miR-520a*, miR-525, and miR-526a) is a characteristic phenomenon of established preeclampsia.

## Figures and Tables

**Table 1:** Correlation between Doppler ultrasonography parameters and C19MC microRNAs expression levels and plasmatic concentrations.

	Spearman's rank correlation											
	Absolute quantification						Relative quantification					
	A. umbilicalis PI		Middle cerebral artery PI		Cerebroplacental ratio		A. umbilicalis PI		Middle cerebral artery PI		Cerebroplacental ratio	
	*ρ*	*P*	*ρ*	*P*	*ρ*	*P*	*ρ*	*P*	*ρ*	*P*	*ρ*	*P*
miR-516-5p	−0.023	0.845	**−0.393**	**0.005**	−0.219	0.125	−0.040	0.736	**−0.307**	**0.030**	−0.190	0.183
miR-517*	0.032	0.791	**−0.328**	**0.020**	−0.215	0.131	0.073	0.542	**−0.288**	**0.041**	−0.269	0.060
miR-518b	−0.025	0.834	−0.152	0.269	−0.074	0.606	−0.019	0.872	−0.220	0.120	−0.167	0.242
miR-520a*	0.092	0.441	**−0.314**	**0.027**	**−0.261**	**0.050**	0.085	0.477	**−0.339**	**0.017**	**−0.339**	**0.018**
miR-520h	0.016	0.893	−0.238	0.093	−0.178	0.214	0.038	0.748	−0.256	0.071	−0.219	0.126
miR-525	0.009	0.941	**−0.358**	**0.011**	−0.190	0.183	−0.028	0.812	**−0.357**	**0.012**	−0.185	0.194
miR-526a	0.156	0.196	**−0.304**	**0.032**	**−0.340**	**0.017**	0.131	0.277	**−0.286**	**0.043**	**−0.329**	**0.021**

The association between the plasmatic concentration (absolute quantification) and gene expression levels (relative quantification) of C19MC microRNAs and Doppler ultrasonography parameters such as arteria umbilicalis pulsatility index, middle cerebral artery pulsatility index, and cerebroplacental ratio.

PI: pulsatility index; *ρ*: Spearman's correlation coefficient; *P*: level of significance; bold font: statistically significant results.

**Table:** (a)

microRNA	miR-516-5p	miR-517*	miR-526a	miR-525	miR-520a*
Number of predicted target genes	349	179	212	340	352

	Unique target genes				Shared with miR-525

	CCR2	FAS	BCAP29	TOX	ACVR2B
	CD109	IL6ST	CD24		AHSA2
	CD1A	IL9R	CD302		ATRN
	DNAJC25	IRAK3	CFLAR		CD2
	FLT1	LILRA2	DNAJC21		CD300LB
	IL17RE	MTDH	HSP90AA1		CD46
	IRAK1	PAPPA	IGFBP1		CD93
	LILRB5		TLR2		HSF5
	PDCD6IP		TNFRSF19		IGF1R
	SOCS2		TNFSF15		IL10RA
			TRAF6		MMD2
					PPARA
					TLR7
					VSIG4

All the targets were predicted by a bioinformatics tool MirTarget2 using miRDB online database.

**Table:** (b)

Gene official symbol	Gene full name	The role in immune system response
ACVR2B	Activin A receptor, type IIB	Activins belong to the TGF-*β* superfamily

AHSA2	AHA1, activator of heat shock 90 kDa protein ATPase homolog 2 (yeast)	Hsp90 is an inducible molecular chaperone protecting stressed cells

ATRN	Attractin	Involvement in initial immune cell clustering during inflammatory responses that may regulate the chemotactic activity of chemokines

BCAP29	B-cell receptor-associated protein 29	Involvement in CASP8-mediated apoptosis

CCR2	Chemokine (C-C motif) receptor 2	Binds monocyte chemoattractant protein-1 involved in monocyte infiltration during inflammation

CD109	CD109 molecule	Encodes GPI-linked glycoprotein that negatively regulates signaling of TGF-*β*

CD1A	CD1a molecule	Encodes glycoproteins structurally related to MHC proteins mediating the presentation of lipid and glycolipid antigens

CD2	CD2 molecule	A surface antigen of thymocytes, T, and NK cells

CD24	CD24 molecule	Encodes a sialoglycoprotein expressed on mature granulocytes and B cells

CD300LB	CD300 molecule-like family member b	A nonclassical activating receptor of the Ig superfamily expressed on myeloid cells

CD302	CD302 molecule	A C-type lectin receptor involved in cell adhesion, migration, endocytosis, and phagocytosis

CD46	CD46 molecule, complement regulatory protein	Has cofactor activity for inactivation of complement components C3b and C4b by serum factor I

CD93	CD93 molecule	Involvement in intercellular adhesion and in the clearance of apoptotic cells

CFLAR	CASP8 and FADD-like apoptosis regulator	Regulator of apoptosis structurally similar to caspase-8

DNAJC21	DnaJ (Hsp40) homolog, subfamily C, member 21	A molecular chaperone protein protecting against cellular stress

DNAJC25	DnaJ (Hsp40) homolog, subfamily C, member 25	A molecular chaperone protein protecting against cellular stress

FAS	FAS cell surface death receptor (FAS)	Plays a central role in regulation of programmed cell death

FLT1	fms-related tyrosine kinase 1	A member of vascular endothelial growth factor receptor (VEGFR) playing an important role in angiogenesis and vasculogenesis

HSF5	Heat shock transcription factor family member 5	A transcriptional activator of heat shock genes

HSP90AA1	Heat shock protein 90 kDa alpha (cytosolic), class A member 1	An inducible molecular chaperone protecting stressed cells

IGF1R	Insulin-like growth factor 1 receptor	Antiapoptotic agent enhancing cell survival

IGFBP1	Insulin-like growth factor binding protein 1	Prolongs the half-time of IGFs in plasma that regulate cell growth and development

IL10RA	Interleukin 10 receptor, alpha	Involvement in inhibition of the synthesis of proinflammatory cytokines

IL17RE	Interleukin 17 receptor E	Participation in MAPK pathway

IL6ST	Interleukin 6 signal transducer (gp130, oncostatin M receptor)	A signal transducer shared by IL-6, LIF, and oncostatin M

IL9R	Interleukin 9 receptor	Mediates IL-9 effects like stimulation of cell proliferation and prevention of apoptosis

IRAK1	Interleukin-1 receptor-associated kinase 1	Responsible for IL-1 induced upregulation of the transcription factor NF-kappa B

IRAK3	Interleukin-1 receptor-associated kinase 3	Functions as a negative regulator of Toll-like receptor signaling

LILRA2	Leukocyte immunoglobulin-like receptor, subfamily A (with TM domain), member 2	An activatory cell-surface receptor expressed on monocytes, B cells, dendritic, and NK cells

LILRB5	Leukocyte immunoglobulin-like receptor, subfamily B (with TM and ITIM domains), member 5	An inhibitory cell-surface receptor expressed on immune cells

MMD2	Monocyte to macrophage differentiation-associated 2	Modulates Ras signaling

MTDH	Metadherin	Involvement in HIF-1 alpha mediated angiogenesis and RNA-induced silencing complex and miRNA functions

PAPPA	Pregnancy-associated plasma protein A, pappalysin-1	Involvement in local proliferative processes such as wound healing

PDCD6IP	Programmed cell death 6 interacting protein	Protects against cell death

PPARA	Peroxisome proliferator-activated receptor alpha	Affects the expression of genes involved in cell proliferation, cell differentiation, and in immune and inflammation responses

SOCS2	Suppressor of cytokine signaling 2	A negative regulator of JAK/STAT cytokine signaling pathway

TLR2	Toll-like receptor 2	Plays a fundamental role in activation of innate immunity, stimulates NF-kappa B

TLR7	Toll-like receptor 7	Plays a fundamental role in activation of innate immunity

TNFRSF19	Tumor necrosis factor receptor superfamily, member 19	Interacts with TRAF family members, induces apoptosis by a caspase-independent mechanism

TNFSF15	Tumor necrosis factor (ligand) superfamily, member 15	A cytokine induced by TNF and IL-1 alpha activating NF-kappa B and MAP kinases inducing apoptosis in endothelial cells

TOX	Thymocyte selection-associated high mobility group box	Highly expressed in thymus, the site of development of T cells

TRAF6	TNF receptor-associated factor 6, E3 ubiquitin protein ligase	Functions as a signal transducer in the NF-kappa B pathway, activates Ikappa B kinase in response to proinflammatory cytokines

VSIG4	V-set and immunoglobulin domain containing 4	A negative regulator of T-cell responses structurally related to the B7 family of immune regulatory proteins
